# *ToxPipe*: integrating AI into toxicoinformatics

**DOI:** 10.3389/ftox.2026.1907845

**Published:** 2026-10-23

**Authors:** Parker K. Combs, Trey O. Saddler, Amlan Talukder, Jonathon F. Fleming, Jeremy N. Erickson, David M. Reif, Charles P. Schmitt, Scott S. Auerbach

**Affiliations:** 1 Predictive Toxicology Branch (PTB), National Institute of Environmental Health Science (NIEHS), National Institutes of Health (NIH), Durham, NC, United States; 2 Axle Informatics, North Bethesda, MD, United States; 3 Dynanet Corporation, Elkridge, MD, United States; 4 Office of Data Science (ODS), NIEHS, NIH, Durham, NC, United States; 5 22nd Century Technologies, McLean, VA, United States; 6 GAP Solutions, Herndon, VA, United States

**Keywords:** artificial intelligence (AI), large language model (LLM), model context protocol (MCP), open-source, retrieval augmented generation (RAG), toxicoinformatics, toxicology

## Abstract

*ToxPipe* is a modular, fully open-source software stack designed to accelerate toxicological data analysis and decision-making by integrating large language model (LLM) capabilities with workflow, monitoring, and user-interface services. *ToxPipe* leverages existing open-source technologies like LibreChat, LiteLLM, and Langflow to provide centralized and streamlined access to different LLMs for end users and developers and logging and auditing of LLM use for organizations. *ToxPipe* also allows for open-source/local model support. While the *ToxPipe* ecosystem also supports advanced AI functions such as MCP server integration, RAG and literature search, and agent creation, we did not observe clear benefits using these configurations relative to the GPT-5.5 baseline assertion coverage scores. *ToxPipe* is available as a series of Docker containers for rapid and reproducible deployment.

## Introduction

The field of toxicology increasingly relies on vast, diverse datasets, such as high-throughput screening data, omics assays, and regulatory text corpora, to assess chemical risks, identify hazardous exposures, and explore biological responses (Thomas et al., 2013; Krewski et al., 2020). This growth in publicly available, heterogeneous toxicological data has created a pressing need for more efficient tools to manage, analyze, and interpret large corpora of complex information (Tariq et al., 2024; Luechtefeld and Hartung, 2025). Traditional approaches often struggle with the volume and variety of available data, leading to inefficiencies in research and risk evaluation processes (Gwinn et al., 2017). There is a clear space for the implementation of new AI-based strategies that are designed from the ground up for efficient and reliable toxicological analysis rather than simply emulating a human researcher’s capabilities (Luechtefeld and Hartung, 2025).

## Background

Over the past decade, significant strides have been made in applying artificial intelligence (AI) to toxicology, particularly in integrating diverse data sources and enabling advanced data-driven insights (Kleinstreuer and Hartung, 2024). Additionally, progress has been made in using AI models to automate visual observation and analysis to enable high-throughput screening for an otherwise tedious manual process (Wang et al., 2024). Toxicological AI tools are also being explored as cheaper, faster, and more consistent alternatives to traditional *in vivo* animal studies (Hartung, 2023; Zhang et al., 2025).

Despite these promising findings, existing AI methods face a series of challenges. Platforms like the U.S. Environmental Protection Agency’s (EPA) Toxicity Forecaster (ToxCast) have pioneered the use of high-throughput screening (HTS) data to predict potential toxicological effects (Kim and Choi, 2025). However, integrating these HTS resources with non-standard, highly specialized, or *in vivo* datasets is challenging due to inconsistent reporting and/or limited metadata (Daniel et al., 2022; Kim and Choi, 2025). Toxicological models based on quantitative structure-activity relationships (QSAR) have been developed to predict chemical toxicity but often struggle to incorporate data from a wide array of experimental and observational sources (Alves et al., 2021). Deep learning models, although powerful, are also often difficult to interpret (Hartung, 2023; Zhang et al., 2025). The underlying datasets powering these AI models are also often not standardized and missing domain-specific data, making it difficult to make accurate predictions about novel examples (Hartung, 2023; Zhang et al., 2025).

One promising recent development in AI is large language models (LLMs). LLMs, in particular, offer unprecedented capabilities for data curation, protocol generation, literature mining, and explanation of complex mode-of-action hypotheses (Lu et al., 2025). Several studies have explored the use of LLMs to handle toxicological datasets, demonstrating their utility in areas such as chemical risk assessment and biological pathway analysis (Kattamreddy and Chinnam, 2025; Ge et al., 2025). The advent of fully capable, accessible AI systems such as AutoGPT (Significant Gravitas, 2025), JARVIS (Shen et al., 2023) and BabyAGI (Nakajima, 2024) have sparked significant interest in the potential for autonomous AI systems in science. The potential for applications tailored to specific fields is characterized by examples such as ChemCrow for chemistry (Bran et al., 2023) and E-KELL for emergency response (Chen et al., 2024). Additionally, recent advancements in retrieval-augmented generation (RAG) for LLMs have provided new opportunities for synthesizing large datasets by combining the power of LLMs with data retrieval systems (Mikulić et al., 2025). RAG systems allow for enhanced context retrieval, drawing relevant information from extensive databases to enrich the outputs of AI models (Gargari and Habibi, 2025). Studies have demonstrated the potential of RAG-based approaches in generating more evidence-supported predictions and detailed summaries in fields such as biomedical research (Liu et al., 2025). Another promising development in providing additional context to models is the Model Context Protocol (MCP), which is an open-source framework developed by Anthropic to facilitate more consistent and scalable communication between LLMs and external resources (Anthropic, 2024). Finally, multimodal and multi-agent systems like EpidemIQs integrate the above technologies into workflows comprising separate AI agents to simulate a team of scientists working together with certain tasks being delegated to the agent specialist most equipped to handle them (Samaei et al., 2026). Such a pipeline is robust to different data types and domains and allows for autonomous or semi-autonomous end-to-end processing and analysis of complex problems (Samaei et al., 2026).

However, current LLM approaches often lack flexibility, scalability, and customization, especially in sensitive fields like toxicology where data privacy and integrity are paramount. There is a growing recognition that customized, domain-specific AI platforms are necessary to meet the unique demands of toxicological research and decision-making (Alam et al., 2025). Some areas of toxicology are poorly represented in scientific literature; thus, AI models may be prone to hallucination when queried about them. Additionally, AI models are often trained on older data and do not possess knowledge of recently generated or published data.

To address these needs, we have engineered *ToxPipe*, an open-source, container-oriented software stack that unifies tools that provide chat-based interaction, workflow composition, model governance, budget management, data provision, and telemetry. *ToxPipe* explores recent advancements in computational toxicology, offering a platform that combines AI, cloud computing, and natural language processing to facilitate toxicological data exploration. By leveraging retrieval-augmented generation (RAG) and agentic workflows, *ToxPipe* allows users to interact with up-to-date, complex datasets in real time through natural language instructions, making it possible to efficiently integrate, summarize, and interpret toxicological information. *ToxPipe*’s support of models hosted through Ollama allows scientists to use locally hosted, privately managed models. However, this capability does not fully guarantee data privacy and proprietary or sensitive data should only be uploaded to an access-controlled deployment that is configured and monitored to prevent routing to a third-party model provider. *ToxPipe*’s customizable design and use of open-source tools allow users and organizations to deploy secure models with functionality tailored to their needs.

This manuscript describes the design and evaluation of *ToxPipe*’s capabilities in toxicological data integration, specifically in the context of chemical toxicity and toxicological reports. The platform’s primary objective is to streamline toxicological research by enabling efficient querying and interpretation of multiple data streams through AI-driven methods such as RAG, literature searching, and agentic workflows. The study also includes an assessment of *ToxPipe*’s performance in handling specific toxicological tasks, such as answering domain-specific questions using natural language instructions. It is important to clarify that the purpose of this work is not to recommend specific regulatory changes or make risk assessment decisions; rather, this study is an evaluation of an LLM’s ability to generate a response and use external resources to a toxicologically-relevant prompt before and after equipping it with access to additional, domain-relevant resources and tools.

This study serves as an exploratory technical demonstration and seeks to investigate the following key research question: when given access to external data sources via RAG and MCP, can a model/agent provided by *ToxPipe* dynamically select and use those sources to answer complex toxicological questions and improve upon basic AI model outputs? Specifically, we examine generator model responses on benchmark strata with higher and lower assertion coverage.

## Methods

The *ToxPipe* ecosystem comprises an inventory of interconnected, open-source AI tools that serve models to process and respond to toxicological queries. Figure 1 shows a high-level overview of ToxPipe's connected components and datasources and how they interact with each other. We designed a multi-tiered, LLM-as-a-judge approach to assess GPT-5.5’s performance across different levels of complexity, focusing on 20 carefully selected chemicals. These chemicals were chosen to represent a spectrum of available toxicological information, ranging from well-documented compounds to novel substances with minimal data. We developed multiple sets of questions comprising general toxicology queries applicable to all chemicals, specific questions targeting National Toxicology Program (NTP) reports (NTP, 2024), and questions derived from various American Board of Toxicology (ABT) examinations. The work described in this section is not necessarily meant to compare models from different providers (as these comparisons will likely become outdated as new models are released) or determine whether a model is suitable for regulatory use. Rather, we present a technical characterization of how our group chose to evaluate model performance before and after integrating *ToxPipe*-specific resources. This evaluation method can be used and adjusted as needed by other groups looking to set up their own deployments of ToxPipe.

**FIGURE 1:**
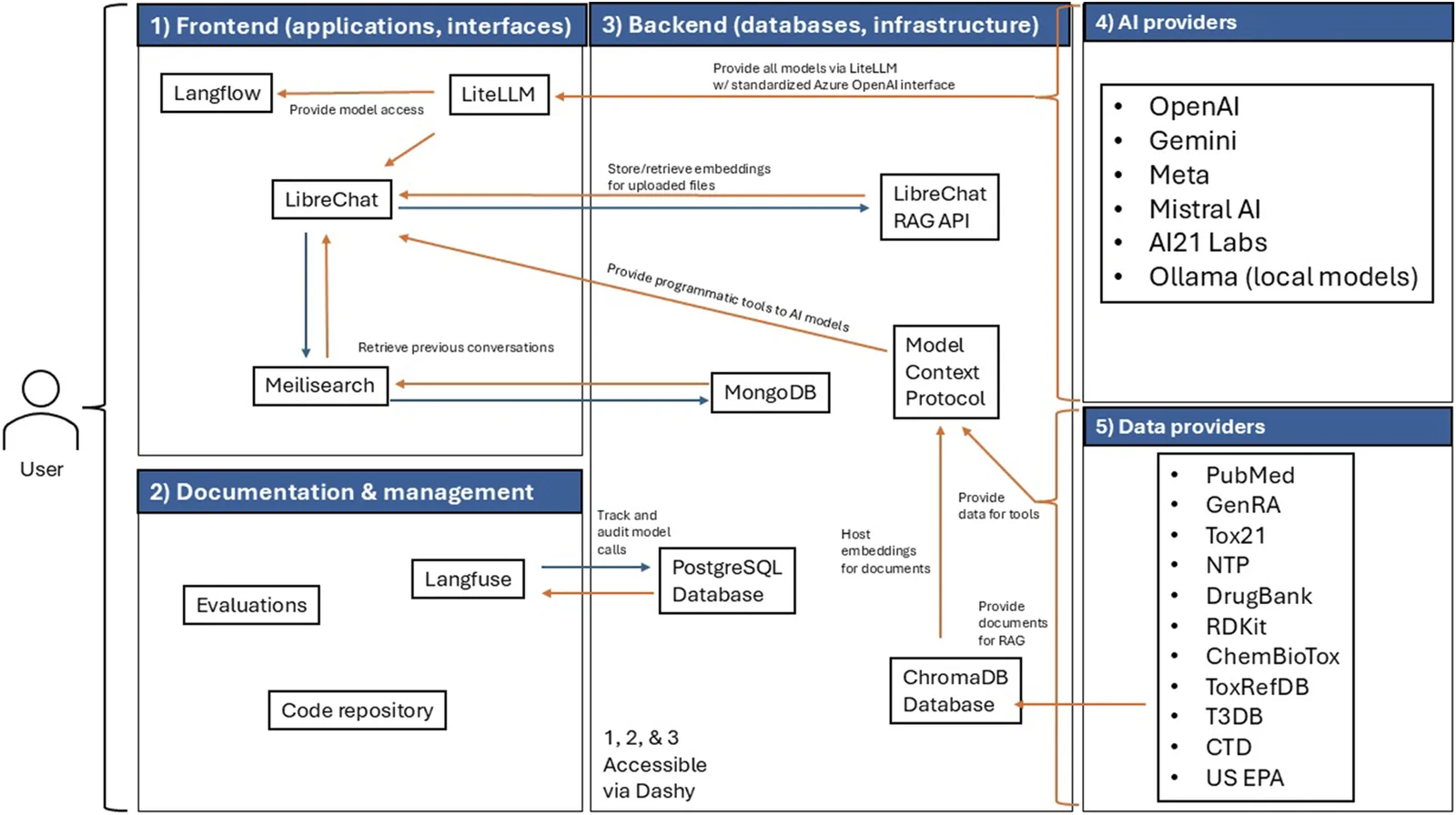
A conceptual overview of the *ToxPipe* ecosystem.

### Implementation stack

To ensure data privacy and enable customized model interactions, we developed a locally-deployable infrastructure utilizing several key components including:

- LibreChat - a ChatGPT-like web platform for conversing and interacting with multiple AI models/agents (Avila, 2025). LibreChat also allows for automatic tool generation when supplied an OpenAPI specification, file upload, and MCP server integration (Avila, 2025).
- LiteLLM - a proxy service for standardizing LLM API calls across different providers (Berri AI, 2025). Instead of integrating each model provider independently, all LLM calls are written against the Azure OpenAI API and routed through LiteLLM, allowing for a single user account to access many different models. This approach simplifies developer onboarding for novel use cases and provides administrators with fine-tuned controls on model usage and budgeting (Berri AI, 2025).
- Langfuse - a web platform for enhanced logging and tracing of model interactions including token usage, latency, tool calls, and other metrics that are crucial in LLM-based development (Langfuse, 2025).
- Ollama - a platform for hosting AI models locally (Ollama, 2025b). Ollama models run entirely locally on a machine and do not interface with or send data to a remote provider, which provides more control over model access and data sharing. However, local models demand hardware requirements such as sufficiently robust graphics processing units (GPUs), and there are only a limited number of models available via Ollama that may have reduced functionality compared to their proprietary alternatives (Ollama, 2025b). For example, Ollama supports only the open-weight version of OpenAI’s GPT models, GPT-OSS (Ollama, 2025a).
- Dashy - a web-based dashboard that provides links to all of *ToxPipe*’s component services and resources as well as uptime monitoring and other administrative views (Sykes, 2025).
- Langflow - a web interface for creating AI agent workflows visually, without the use of code (Langflow-AI, 2025). Langflow allows users to quickly prototype workflows ranging from simple, linear processes to complex, branching, multi-agent flows that integrate APIs, search engines, etc. (Langflow-AI, 2025)

*ToxPipe* also provides a separate suite of software tools that can be used by AI models. We provide a dedicated model context protocol (MCP) server, *ToxPipeMCP*, which is built on the FastMCP (v2.13.3) framework (PrefectHQ, 2026) and may be deployed separately from the rest of the *ToxPipe* ecosystem and connected to models from *ToxPipe* or other LLM deployments. *ToxPipeMCP* connects to the ChemBioTox API to provide LLMs with tools that access toxicological data curated from reputable databases such as the Comparative Toxicogenomics Database (CTD) and Toxicity Reference Database (ToxRefDB) for the over 1 million chemicals in the EPA’s CompTox Chemicals Dashboard (EPA, 2025; Williams et al., 2017). *ToxPipeMCP* also supports scientific literature search capabilities. First, it provides retrieval-augmented generation (RAG) from text embeddings of documents provided by the United States National Toxicology Program (NTP) at https://ntp.niehs.nih.gov/publications. Additionally, it can perform a literature search of relevant manuscripts on PubMed. Providing these external data sources to the models in *ToxPipe* attempts to fill in both domain-based and temporal gaps in the models’ training data.

### Deployment and sharing

The public code repository for *ToxPipe* and related applications can be found at https://doi.org/10.22427/DATA-500-110-003-000-0. Each of *ToxPipe*’s constituent services is fully configured in separate Docker containers, which are linked with Docker Compose for easy management. *ToxPipe* is offered as open-source software and is licensed under the MIT license.

### Additions to base LLMs

#### Expanded model access

*ToxPipe* provides access to additional models not formally offered to or supported by the United States government. Our internal deployment has been tested with models from the following providers:

- AI21 (Jamba models)
- Amazon (Nova models)
- Anthropic (Claude models)
- Cohere (Command R Plus model)
- Google (Gemini models)
- Meta AI (Llama models)
- MistralAI (Mistral models)
- OpenAI (GPT and o models)

All of these models are accessible from the LiteLLM component of *ToxPipe* using the Azure OpenAI API. All of a user’s allowed models may be accessed with just a single API key per user, eliminating the need for an end user to manage multiple accounts and subscriptions across different providers to use a variety of models. Furthermore, different LLMs have different capabilities: some are incompatible with tools, some take on a more direct (rather than conversational) tone, etc. Therefore, certain workflows may require or benefit from multiple LLMs in their pipelines: *ToxPipe*’s setup allows easy use of multiple different models using a single set of credentials. Additionally, *ToxPipe* can serve locally hosted models (via Ollama) over the LiteLLM API, which may be more appropriate for working with proprietary data. However, it is important to keep in mind that the availability and usage of locally hosted models is not a guarantee of data privacy or security, and care should be taken to not upload any sensitive work or data without proper access control and security protocols in place.

#### Agentic workflows

LLM agents allow a user to define a specific role for a model by providing additional instructions and equipping it with programmatic “tools” to allow the model to pull in external data to consider alongside its training data when formulating a response. Additionally, agents can assess and respond to user queries in iterative steps rather than using the singular, zero-shot method used by base LLMs. This allows an agent to take an action, evaluate its progress toward answering the original prompt, and take further actions as required. Ideally, this enhanced capability would allow AI agents to provide evidence-supported responses when querying a low-assertion stratum. We evaluated the agents to determine whether tool availability and the quality of tool-provided data improved the quality of AI responses and to identify gaps in our own data corpus. The LibreChat interface natively supports the creation and querying of agents (Avila, 2025). Programmatic agents may be created using tools and libraries like LangChain and the Claude Agent SDK in Python.

#### Tools and actions

*ToxPipeMCP*’s tools are snippets of Python code with accompanying descriptions that may be called at an AI agent’s discretion. This allows developers to define arbitrary functionality to be added to a model’s capabilities. *ToxPipe* contains a growing set of tools that allow an AI agent to access curated toxicological data. These tools provide LLMs with access to functions for manipulating and analyzing molecules (provided by RDKit) (Rdkit, 2024) and access to the NIEHS’s ChemBioTox database, which hosts experimental and predicted data for over 1 million chemicals, including predicted data for chemicals that are poorly characterized in the published literature. These tools include functions that produce structured chemical property or behavior data when supplied with a chemical identifier. These tools allow access to attributes such as physical properties (e.g., solubility and molecular weight); associated diseases, genes, and targets; similar chemicals; exposure data; and other health effects. *ToxPipeMCP* tools may also be used with models that are not provided through *ToxPipe*. These tools may be used by agents or invoked by zero-shot or conversational models.

*ToxPipeMCP*’s code may be found at https://doi.org/10.22427/DATA-500-110-003-000-0. *ToxPipeMCP* is offered as open source software and is licensed under the MIT license.

#### RAG search and text embeddings

RAG—providing a database of text embeddings to an AI model for use as additional context when generating a response—allows for more direct control over what information the model has access to and can potentially help the model give more relevant answers to more technical or domain-specific queries. A researcher can curate a corpus of documents to be embedded to fill in any temporal or domain gaps in the model’s training data. *ToxPipe* is capable of performing RAG from text embeddings generated from scientific documents provided by the National Toxicology Program (NTP) (NTP, 2024). These documents comprise various toxicity, technical, and chemical reports: each document was processed according to the process outlined in Figure 2. Using the LlamaIndex framework, we created a pipeline where an instance of GPT-4o extracted document metadata (article title, keywords, a summary, and any questions answered by the document), and an instance of text-embedding-ada-002 generated the text embeddings with 1,536 dimensions (LlamaIndex, 2025). We chunked the documents such that each page of a given document was treated as a separate text node by LlamaIndex up to a maximum chunk size of 1,000. If a given page exceeded this character count, it was split into chunks of size 1,000 with an overlap of 128. The embeddings creation process implemented simple retry logic with fixed backoff where a failed document was re-processed up to ten times with 3 seconds of sleep time in between each attempt. All other parameters were left as their default values. Once all embeddings were created, we stored them in a database using the open-source vector database platform ChromaDB for data persistence and retrieval (Github, 2025). The embeddings database may be downloaded from https://doi.org/10.22427/DATA-500-110-003-000-0: we used the collection name “quickstart.”

**FIGURE 2:**
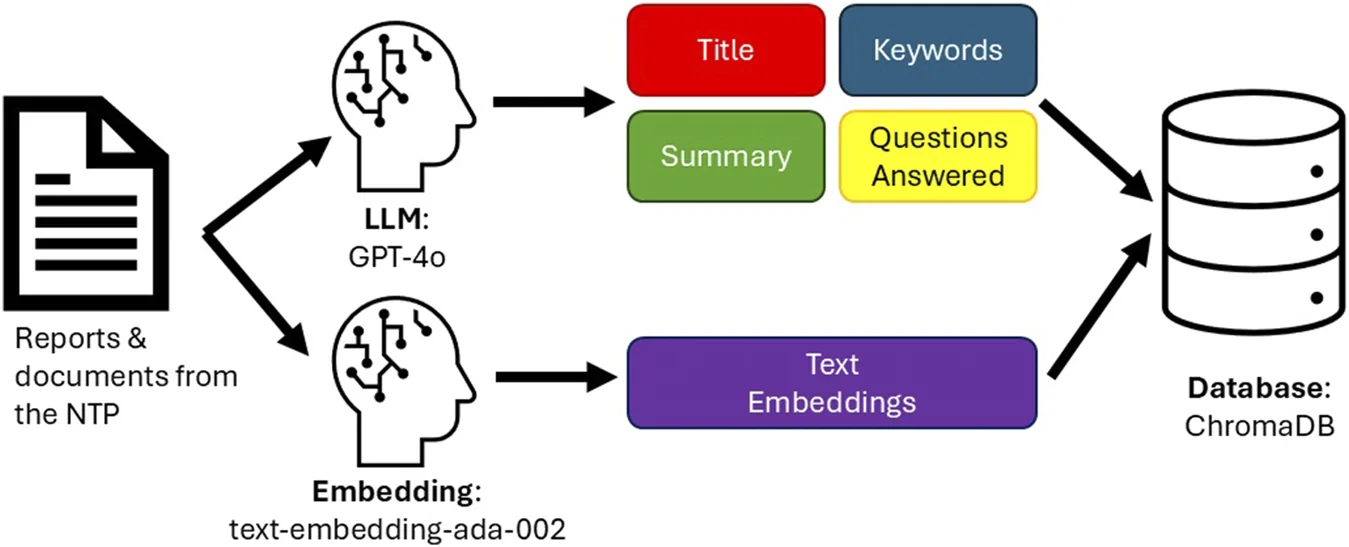
An overview of *ToxPipe*’s document ingestion and text embedding creation process for use in RAG.

RAG functionality using these embeddings is also provided as a tool in the *ToxPipeMCP* server. Both *ToxPipeMCP*’s RAG configuration and our evaluations used the text-embedding-ada-002 model for document retrieval. This retrieval workflow performs a search for relevant data to answer the user’s query by first converting the user’s query into a set of up to ten keywords. The system then retrieves up to five document embeddings associated with keywords relevant to those extracted from the user’s query based on a similarity score threshold of 0.3. Finally, the model synthesizes a response using any retrieved embeddings as context. Optionally, the LLM may be instructed to formulate a response using its training data if it is unable to appropriately answer the user’s query using only retrieved documents. The flexibility and open-source nature of *ToxPipe* means that a user could create an embeddings dataset derived from proprietary or otherwise non-public documents and provide that as an additional RAG resource to their own deployment of *ToxPipe*; however, this should only be done in a fully-local, access-controlled environment.

#### Literature search

Also accessible from the *ToxPipeMCP* server is a custom implementation of an LLM-powered academic literature search. *ToxPipe* uses an AI model to extract keywords from the user’s query. These keywords are passed as search input to the National Center for Biotechnology Information’s (NCBI’s) E-utilities API at https://eutils.ncbi.nlm.nih.gov to search for relevant manuscripts in PubMed. If the search returns results, the *ToxPipe* API downloads those papers, extracts their texts, and uses the LLM to generate a 1-2 paragraph summary of each before returning them to the user. As with the RAG search functionality, the literature search is also used to supplement the AI model’s training data with scientific, domain-specific, curated data.

#### Portability and reproducibility

The entire *ToxPipe* ecosystem is containerized using Docker and thus runs as a series of interconnected services. The *ToxPipe* repository may be cloned and its ecosystem configured according to an end user’s specifications. *ToxPipeMCP* may also be run as a standalone service to provide *ToxPipe*’s data component to other LLM systems outside of *ToxPipe*.

### Documentation

*ToxPipe* provides an evolving selection of documentation, available at https://github.com/NIEHS/ToxPipe/tree/Deployment/docs, with guidance on setting up local, customizable deployments of the *ToxPipe* ecosystem and connecting its models to popular third-party tools like Claude, OpenCode, Positron, and Copilot for Visual Studio Code.

### Evaluation

To evaluate the *ToxPipe* enhancements, we submitted a selection of toxicological questions to independent instances of OpenAI’s GPT-5.5 model and compared their responses to a predefined set of assertions corresponding to a given question. These assertions were developed by subject matter experts within the NIEHS to evaluate the LLM’s ability to select among multiple-choice options, decide on and use tools, and extrapolate on incomplete data. Each prompt was submitted to GPT-5.5 three times to account for any potential variance when answering. Additionally, each run was performed independently of all other runs with no prior history or context outside of a base system prompt to ensure that earlier runs do not influence later runs. We conducted three independent experimental rounds of the generation-evaluation process to examine any stochasticity in model processing or evaluation. We chose to use GPT-5.5 for responding to queries as, from our testing, it produced analyzable final records in the evaluated base, RAG, and MCP configurations. Runs that produced errors or did not run to completion were rerun until they produced final records; however, we did not retain a log of transient failures and retries. Therefore, GPT-5.5’s response completeness does not necessarily indicate a lack of mechanical error.

We used the LLM-as-a-judge methodology to evaluate a given model response to determine how semantically similar its content was to one or more of the assertions present for the given prompt. Each generated response was evaluated once by each of GPT-5.5, Gemini 3.5 Flash, and Claude 4.5 Haiku. Within a given round, three responses were generated independently per prompt. Each prompt was evaluated a total of 9 times per round and 27 times in total across three rounds. Human review was used in select cases in development to ensure the model pipeline was properly running, but the full evaluation process was entirely automated and determined by the three evaluator models. The evaluation process’s code and methodology are formally documented at https://doi.org/10.22427/DATA-500-110-003-000-0 (Talukder et al., 2026). We included the Gemini and Claude evaluator models to avoid reliance on the same evaluator model as the generator model, but this method does not eliminate evaluator bias.

We used high-reasoning mode for the GPT-5.5 instance responding to the queries across all runs to help the model work through any complex data fetching and multi-step, multi-tool workflows. We used GPT-5.5 with low reasoning during the evaluations, as we found it to be much faster and sufficient for judging the matching of assertions.

The LLM was evaluated at three levels:

1. Level 1: base model—GPT-5.5 was queried without additional context or information to assess its baseline knowledge and capabilities in toxicology. Prompting at this level was performed in a zero-shot fashion: the model was given a single prompt to respond to without any previous context.
2. Level 2: base model + RAG—GPT-5.5 was provided with additional information from embeddings of toxicological documents from the NTP. Unlike level 1, where the model operates on pretrained knowledge alone, level 2 can potentially provide the AI with access to up-to-date, domain-specific information. It is important to note that evaluations at this level are meant to evaluate our specific RAG implementation and not RAG as a whole.
3. Level 3: agent with tools—This level integrated agentic workflows, where the model acts in an iterative, multi-step fashion instead of in a context-limited, zero-shot way. Instead of retrieving and integrating data in a single step, an agent continuously refines its answer by actively selecting and calling relevant code tools that interface with curated data sources when formulating responses. This agent-based framework allowed for multi-step reasoning, which is better equipped for answering more complex queries and assessing more advanced problem-solving capabilities in toxicological contexts. This process permits iterative retrieval and tool use, enabling the GPT-5.5 model to explore a variety of data sources and refine its conclusions through multiple iterations. RAG functionality was also provided as a callable tool to the GPT-5.5 model at this level, although its use was not forced. MCP also allows for more direct control over the model’s behavior, as a developer has full control over how a given MCP tool is laid out; that is, a developer can define the exact steps they want a model to take if it decides to call the tool. We suspect that MCP tools can help with specificity-related problems, such as sanitizing the names and IDs of chemicals to ensure they resolve to the correct chemical before fetching data for them.

Four categories of questions were provided to each level:

1. ABT Q/A prompts — 474 multiple-choice questions pulled from American Board of Toxicology (ABT) exams issued from 2000 through 2007 using optical character recognition (OCR) (Talukder et al., 2026). These prompts were included to test the model’s ability to distinguish between multiple possible answers and select the best one(s).
2. Agent-focused Q/A prompts — 10 questions that comprise a mixture of open-ended and multiple choice questions requiring the model to make toxicological assessments when given chemical SMILES strings (Talukder et al., 2026). These prompts were designed to facilitate the use of programmatic tools to find information to be used to generate a response rather than the reliance on semantic similarity or word co-occurrence using the model’s training data.
3. Toxicity type: human prompts — 15 open-ended questions (14 toxicity-system prompts plus toxicokinetic parameters) pertaining to the behavior and toxicological effects of chemicals as observed in humans (Talukder et al., 2026). These questions generally take the form: “List the (toxicity_type) of (chemical) on (species)”. The following toxicity types were used in queries: subacute, chronic, developmental, immune/lymphatic system, reproductive system, nervous system/neurological, digestive system, urinary system, integumentary system, musculoskeletal system, cardiovascular system, endocrine system, respiratory system, hematological system, and additional toxicokinetic parameters (Talukder et al., 2026). These prompts were selected to test the model’s ability to identify and use relevant data from text embeddings extracted from scientific documents and technical reports. These prompts are meant to test the model’s ability to identify assertion-supported information from the high-assertion corpus.
4. Toxicity type: rat prompts — 16 open-ended questions (14 toxicity-system prompts, toxicokinetic parameters, and subchronic toxicity) pertaining to the behavior and toxicological effects of chemicals as observed in rats (Talukder et al., 2026). These questions generally take the form: “List the (toxicity_type) of (chemical) on (species)”. The following toxicity types were used in queries: subacute, chronic, developmental, immune/lymphatic system, reproductive system, nervous system/neurological, digestive system, urinary system, integumentary system, musculoskeletal system, cardiovascular system, endocrine system, respiratory system, hematological system, subchronic, and additional toxicokinetic parameters (Talukder et al., 2026). These prompts were selected to test the model’s ability to identify and use relevant data from text embeddings extracted from scientific documents and technical reports. These prompts are meant to test the model’s behavior in the lower-assertion stratum.

These questions are not intended to constitute a representative or comprehensive sample and conclusions drawn from the LLM’s responses to them would be inappropriate for regulatory use. Conclusions about a chemical’s behavior should not be drawn from the evaluations in this study. Rather, this is a small but varied subset of questions, potentially helpful for screening tasks, that we chose to explore and demonstrate how *ToxPipe*’s data and tool additions may affect an LLM’s ability to respond. These questions were formulated to explore different prompt and expected answer formats as well as the model’s performance across domains with different levels of documented information.

In the toxicity type prompts, we selected 20 chemicals to include in the queries (Table 1). These chemicals were selected to cover a range of domain knowledge: chemicals higher on the list like Aspirin and Paracetamol are well-characterized, chemicals 1-15 have rat toxicity data, chemicals 1-11 have human toxicity data, and chemicals 16-20 have limited overall toxicity data for rats and humans. Additionally, only combinations of chemical/toxicity type with a known association (i.e., an established ground truth value) were given assertions. Evaluations were still run on chemical/toxicity type pairs without assertions, but they were used only to explore the capabilities of *ToxPipe*, and their scores did not contribute to the overall model scores.

**TABLE 1:** The 20 chemicals used in the toxicity type LLM evaluations.

Number	Chemical name	CASRN
1	Aspirin	50-78-2
2	Paracetamol	103-90-2
3	Metformin	657-24-9
4	Atorvastatin	134523-00-5
5	Prednisone	53-03-2
6	Dioxin	1746-01-6
7	Benzene	71-43-2
8	Arsenite	15502-74-6
9	Methylmercury	16056-34-1
10	Vinylchloride	75-01-4
11	6PPQ (N-(1,3-dimethylbutyl)-N′-phenyl-p-phenylenediamine)	2754428-18-5
12	Phenanthrene	85-01-8
13	TPHP (triphenylphosphate)	115-86-6
14	Lanthanum	7439-91-0
15	Galaxolide	1,222-05-5
16	1,6-Dimethyl-3,4-dihydroisoquinoline	91753-09-2
17	2-Methyl-4-(4-methylphenyl)-2,3-dihydro-1,5-benzothiazepine	74148-62-2
18	N-Cyclopropylmethyl-10,11-dihydro-5H-dibenzo-(a,d)-cyclohepten-5-imine	59864-46-9
19	4-Methyl-1,2-dihydrobenzo [f]isoquinoline	29248-42-8
20	6-Methyl-2,5-diphenyl-6H-1,3,4-thiadiazine	62625-70-1

The LLM was queried with all of the toxicity type prompts (15 for humans, 16 for rats) for each of the selected 20 chemicals, resulting in 300 total prompts for toxicity type: human and 320 total prompts for toxicity type: rat. However, many of these chemical-toxicity type combinations do not have known associations, so only 57 of these prompts were evaluated for the toxicity type: human category and only 12 prompts were evaluated for the toxicity type: rat category. The two toxicity type prompt categories were constructed to evaluate LLM performance on different benchmark strata with different amounts of assertion support. The toxicity type: human prompts were related to well-documented, generic manifestations of human pathologies and were aimed at testing LLM performance in a higher-assertion-coverage benchmark stratum. The toxicity type: rat prompts covered rarer and more obscure pathologies primarily documented in the NTP reports at https://ntp.niehs.nih.gov/publications and were aimed at testing LLM performance in a lower-assertion-coverage benchmark stratum.

#### Scoring

We took the following steps to score each run (Talukder et al., 2026). Note that these steps were repeated across three rounds to account for stochasticity in model output and evaluator judgment:

1. For each prompt, identify assertions—expected keywords or key phrases—that must be present in the generated response for the evaluation to be considered “successful” (Talukder et al., 2026).
2. For each round, run each prompt three times to account for any potential answer variance when generating a response at each level, producing nine responses per prompt per level total (Talukder et al., 2026).
3. Provide each response generated by step 2 to the three evaluator models to perform a semantic similarity check between the content of the model’s response and the predefined assertions for the corresponding prompt (Talukder et al., 2026). This produces nine evaluations per prompt per level within a given round and 27 evaluations across all three rounds.
4. For each evaluator judgment, assign the response partial credit according to the proportion of reference assertions matched semantically (i.e., matching 2/3 assertions would award the model with a score of 0.67 for that evaluation). This produces an intermediate evaluation score. We decided to award partial credit to runs since an “all-or-nothing” approach to model success may not be an appropriate gauge of model capabilities in all use cases (such as exploring a topic in an open-ended way).
5. For a given level, take the average intermediate score across all 3 evaluator models and all 3 runs for a given prompt (i.e., the mean score across 9 runs) to get an overall evaluation score for that prompt. If this score is greater than 0.5, the run is a “pass”; else, it is a “failure.” Figures 3–5 document these pass/fail counts for levels 1, 2, and 3, respectively.
6. Once all prompts have been run and evaluated as outlined above, descriptive means were calculated for each level and prompt category (ABT Q/A, agent-focused Q/A, toxicity type prompts: human, and toxicity type prompts: rat) (Talukder et al., 2026). Therefore, each level has four evaluation scores: one for each prompt category. Table 2 contains the mean evaluation scores across all three rounds of runs, in which the response generation and scoring process was repeated 3 times and the resulting per-round scores were averaged. For each prompt category and level, we first averaged prompt-level scores within each round and then averaged the three round-level means to calculate the “Overall” scores. Note that the “GPT”, “Claude”, and “Gemini” values in each cell in Table 2 refer to the scores generated by the evaluator models, *not* the model used to generate the response to the prompt, as all responses were generated with GPT-5.5. Figure 6 provides a visual guide to this scoring process. We did not calculate confidence intervals because the prompts form a curated benchmark rather than a representative probability sample. Observed numerical differences are descriptive and are not interpreted as reflective of the population.

**FIGURE 3:**
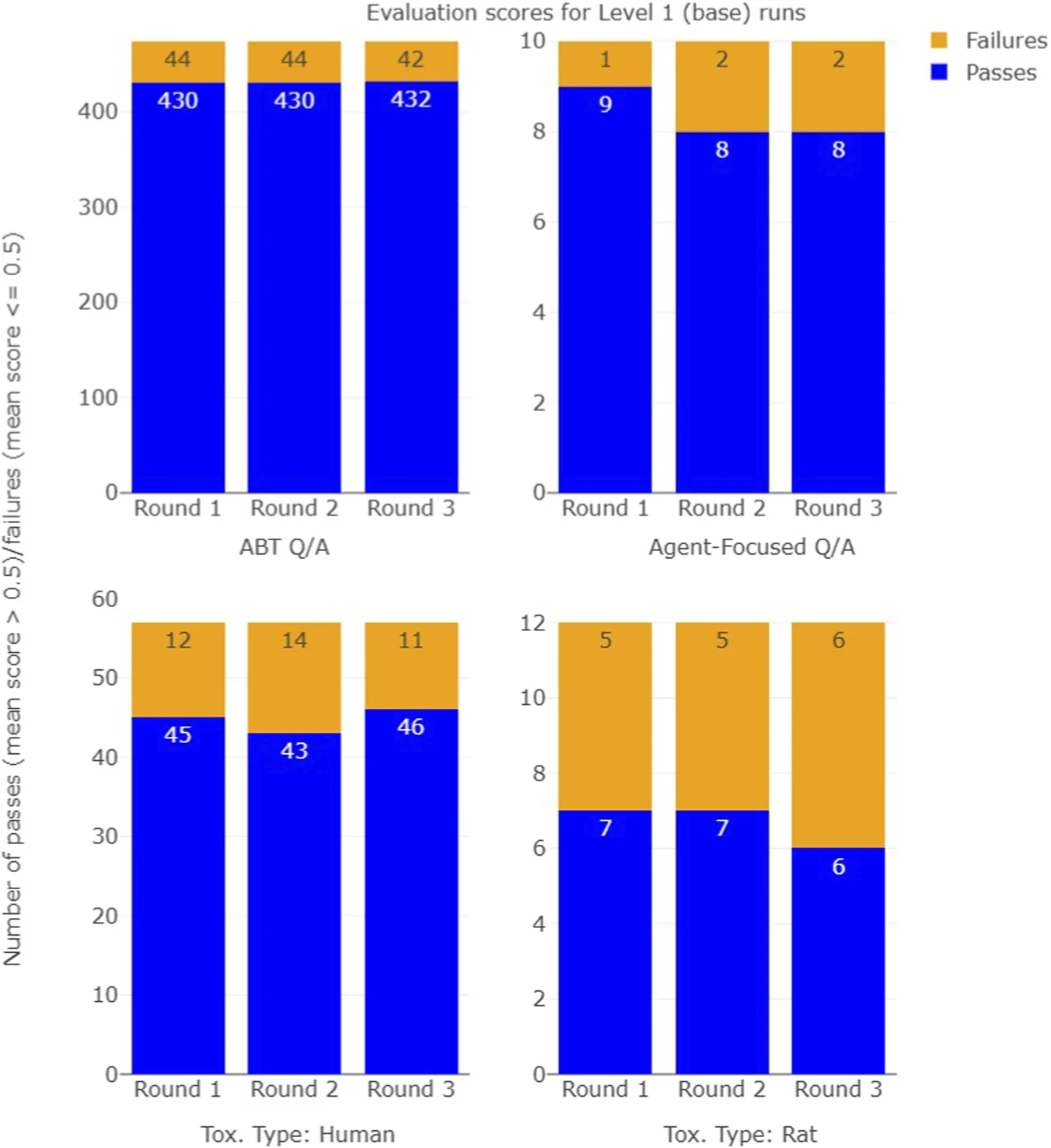
Evaluation scores across all 3 rounds and all 4 categories for the level 1 (base) runs. A “pass” is any evaluation where the mean evaluation score across the 3 runs and 3 evaluator models (9 evaluations per prompt per round) is greater than 0.5 for a given prompt.

**FIGURE 4:**
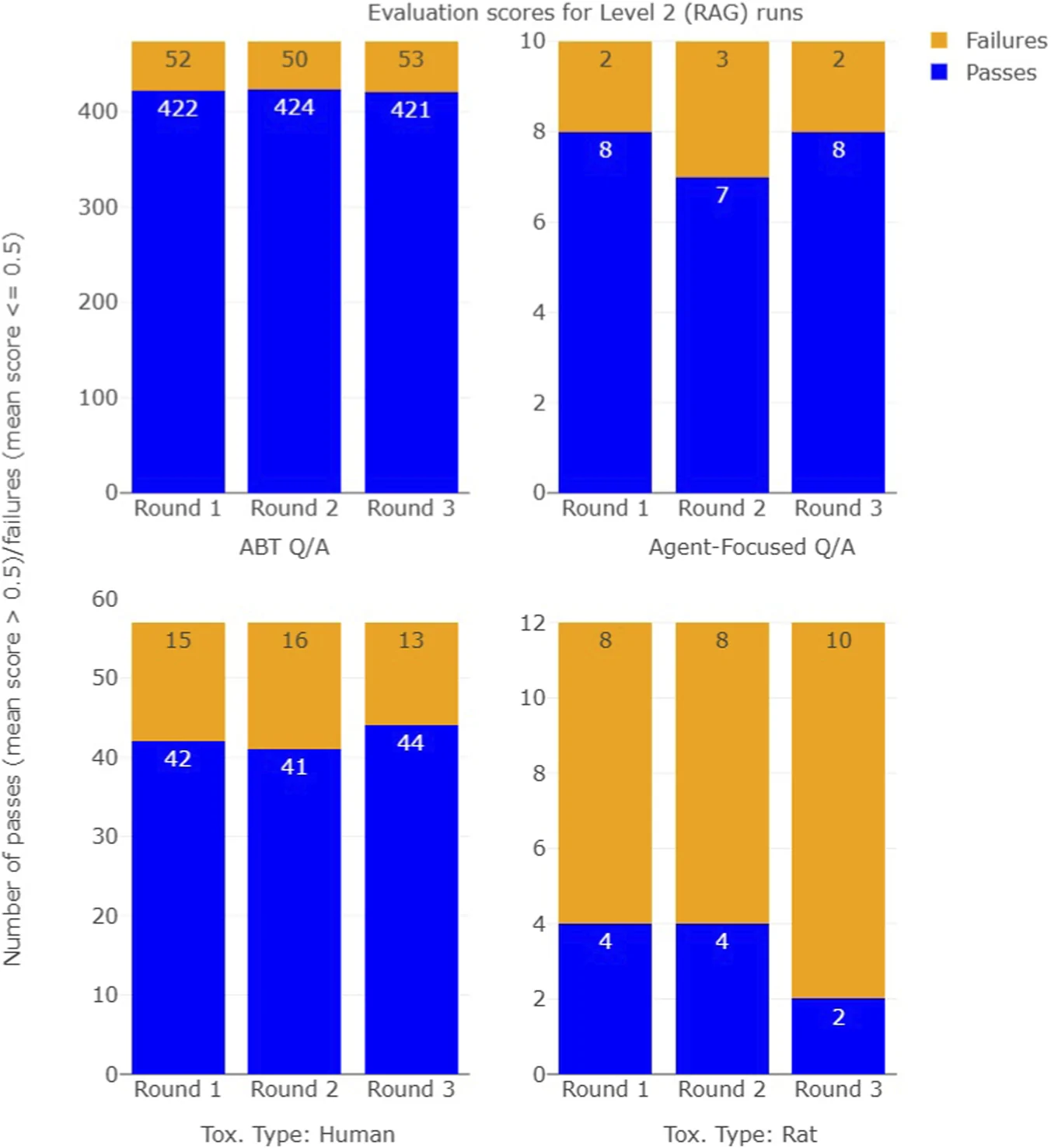
Evaluation scores across all 3 rounds and all 4 categories for the level 2 (RAG) runs. A “pass” is any evaluation where the mean evaluation score across the 3 runs and 3 evaluator models (9 evaluations per prompt per round) is greater than 0.5 for a given prompt.

**FIGURE 5:**
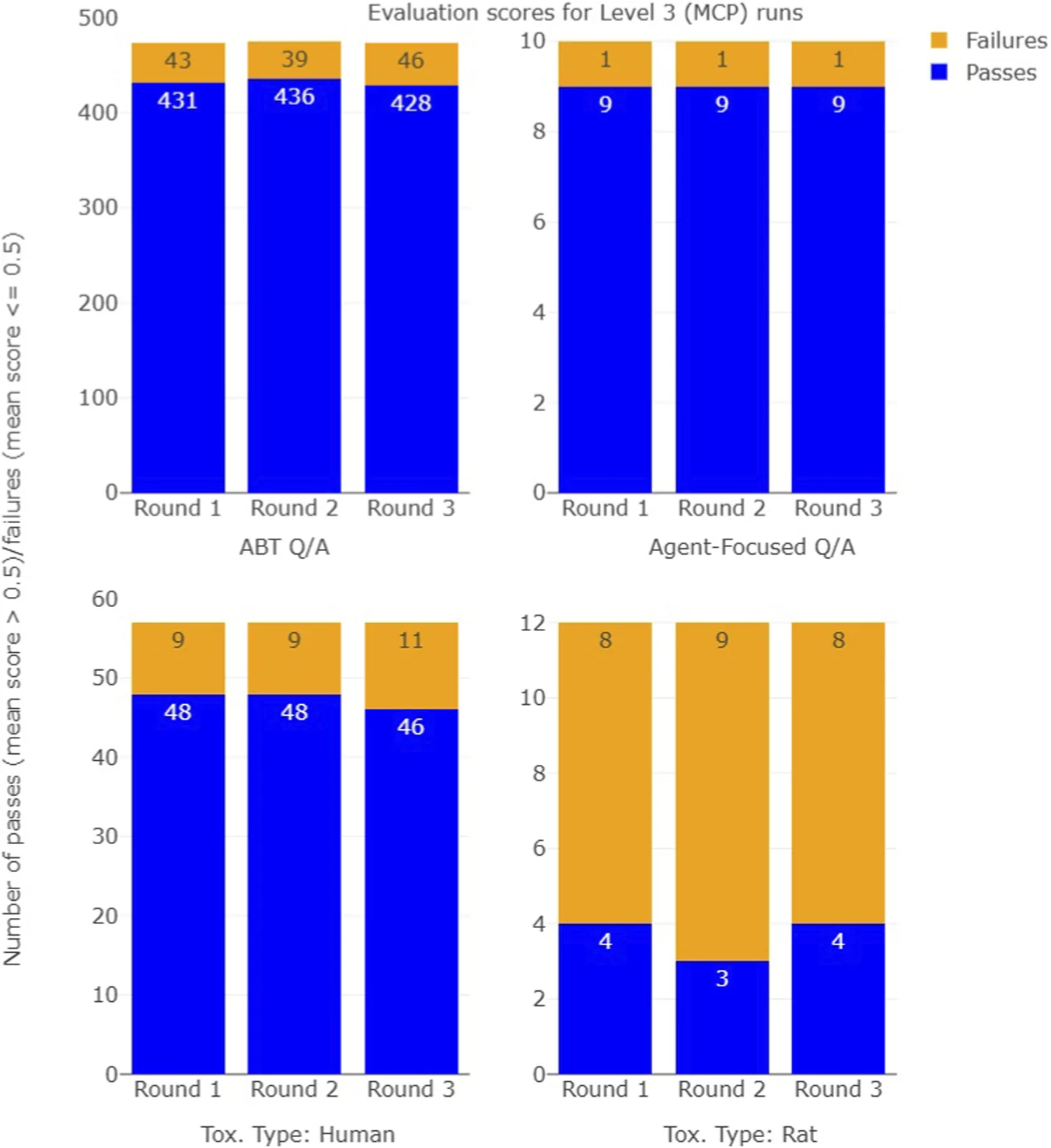
Evaluation scores across all 3 rounds and all 4 categories for the level 3 (MCP) runs. A “pass” is any evaluation where the mean evaluation score across the 3 runs and 3 evaluator models (9 evaluations per prompt per round) is greater than 0.5 for a given prompt.

**FIGURE 6:**
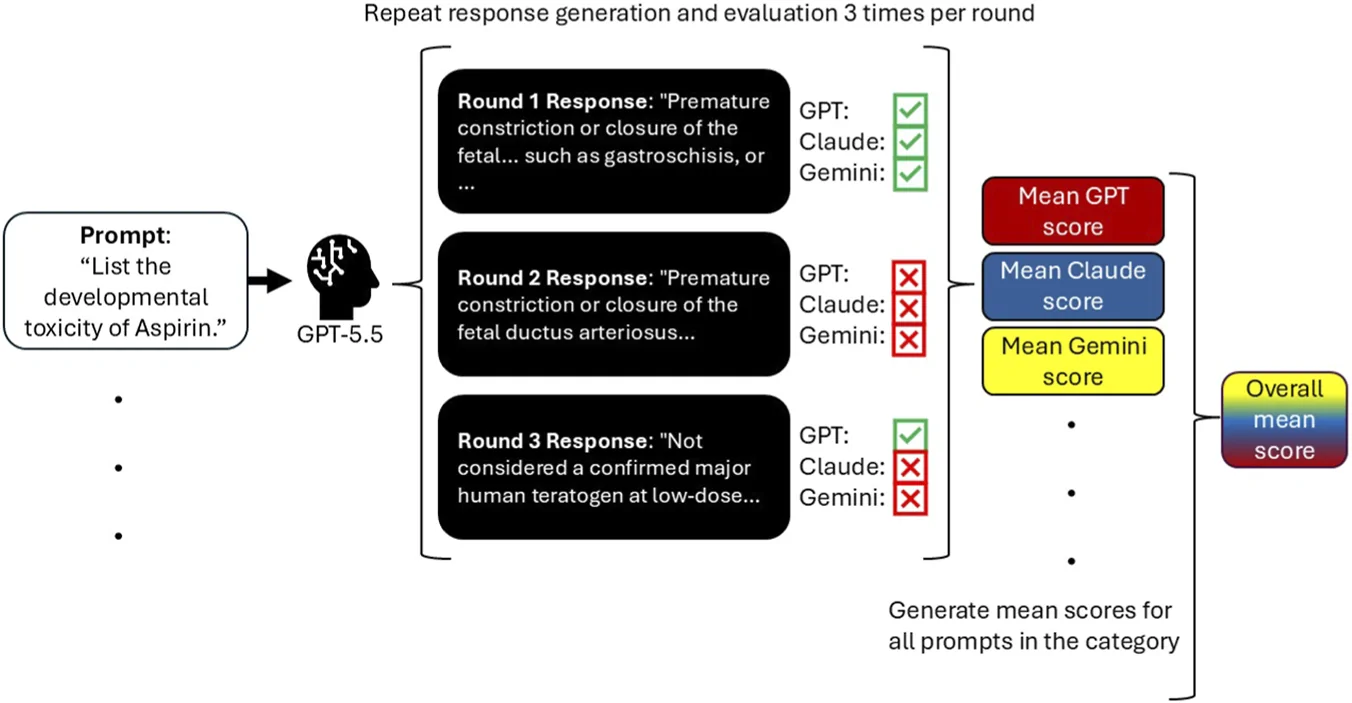
A visual walkthrough of our evaluation and scoring method for *ToxPipe*. For a given prompt, 3 responses are generated at each level. Each response is evaluated once by the GPT, Claude, and Gemini evaluator models, for a total of 9 evaluations per prompt. This process is repeated for each prompt in a given category to get categorical means for the GPT, Claude, and Gemini evaluator models. The overall mean score for the category is then calculated by taking the mean of the 3 categorical evaluator model mean scores. This process is followed at each of the 3 levels.

**TABLE 2:** *ToxPipe* model evaluation scores across all 4 prompt categories and all 3 levels using the 3 evaluator models. For each level and prompt category, the table reports three evaluator-specific means, their overall mean, and their range: “GPT” - average of the intermediate mean scores for the prompt category as scored by GPT-5.5; “Claude” - average of the intermediate mean scores for the prompt category as scored by Claude 4.5 Haiku; “Gemini” - average of the intermediate mean scores for the prompt category as scored by Gemini 3.5 Flash; “Range” - the range of the mean GPT, Claude, and Gemini scores; and “Overall” - mean evaluation score of the mean GPT, Claude, and Gemini scores.

Average statistics across all rounds				
​	Prompt category (number of prompts)			
Score	ABT Q/A (474 prompts)	Agent-focused Q/A (10 prompts)	Tox. type: human (300 prompts)	Tox. type: rat (320 prompts)
Level 1 - base model only				
GPT	0.89 (0.82, 0.91, 0.92)	0.83 (0.90, 0.80, 0.80)	0.81 (0.80, 0.78, 0.84)	0.59 (0.63, 0.59, 0.56)
Claude	0.89 (0.89, 0.89, 0.90)	0.83 (0.90, 0.80, 0.80)	0.75 (0.76, 0.74, 0.75)	0.54 (0.55, 0.50, 0.56)
Gemini	0.91 (0.88, 0.92, 0.92)	0.83 (0.90, 0.80, 0.80)	0.78 (0.78, 0.77, 0.78)	0.55 (0.58, 0.58, 0.49)
Overall	0.89 (0.86, 0.91, 0.91)	0.83 (0.90, 0.80, 0.80)	0.78 (0.78, 0.76, 0.79)	0.56 (0.59, 0.55, 0.54)
Range	0.04 (0.06, 0.02, 0.02)	0.00 (0.00, 0.00, 0.00)	0.06 (0.05, 0.04, 0.09)	0.08 (0.08, 0.09, 0.07)
Level 2 - base model with RAG				
GPT	0.90 (0.89, 0.90, 0.90)	0.77 (0.80, 0.70, 0.80)	0.72 (0.70, 0.72, 0.73)	0.36 (0.36, 0.37, 0.34)
Claude	0.87 (0.86, 0.88, 0.88)	0.79 (0.80, 0.70, 0.87)	0.70 (0.69, 0.69, 0.72)	0.30 (0.34, 0.35, 0.23)
Gemini	0.90 (0.90, 0.90, 0.91)	0.77 (0.80, 0.70, 0.80)	0.71 (0.69, 0.71, 0.72)	0.36 (0.39, 0.37, 0.32)
Overall	0.89 (0.88, 0.89, 0.89)	0.77 (0.80, 0.70, 0.82)	0.71 (0.70, 0.71, 0.72)	0.34 (0.36, 0.36, 0.29)
Range	0.03 (0.04, 0.03, 0.03)	0.02 (0.00, 0.00, 0.07)	0.02 (0.01, 0.03, 0.01)	0.06 (0.05, 0.03, 0.11)
Level 3 - agent with MCP				
GPT	0.91 (0.91, 0.92, 0.91)	0.89 (0.90, 0.90, 0.87)	0.81 (0.80, 0.82, 0.79)	0.37 (0.37, 0.35, 0.38)
Claude	0.89 (0.90, 0.88, 0.89)	0.89 (0.90, 0.90, 0.87)	0.74 (0.73, 0.76, 0.74)	0.36 (0.36, 0.34, 0.38)
Gemini	0.92 (0.92, 0.92, 0.91)	0.89 (0.90, 0.90, 0.87)	0.80 (0.80, 0.79, 0.80)	0.40 (0.43, 0.35, 0.40)
Overall	0.91 (0.91, 0.91, 0.90)	0.89 (0.90, 0.90, 0.87)	0.78 (0.78, 0.79, 0.78)	0.37 (0.39, 0.35, 0.39)
Range	0.03 (0.02, 0.04, 0.03)	0.00 (0.00, 0.00, 0.00)	0.06 (0.07, 0.06, 0.06)	0.04 (0.07, 0.01, 0.03)

It is also appropriate to note the main limitation of this scoring strategy: responses are rewarded for containing the proper assertion(s) but not penalized for containing additional data, even if they do not support the assertion(s).

## Results

Table 2 provides a detailed breakdown of model performance across all 3 levels for each prompt category.

### Base model performance

Figure 3 and Table 2 provide baseline evaluation scores for the base GPT-5.5 model across all 4 prompt categories. The scores we observe here generally follow our expectations. The ABT Q/A runs achieved relatively high scores across all 3 rounds. We can see that the generator model achieved higher scores with less variance across runs on the toxicity type: human prompts compared to the toxicity type: rat prompts using the base model only.

### RAG performance and comparison to base model

Figure 4 and Table 2 outline the RAG model evaluation scores across all prompt categories. We can observe a similar trend in evaluation scores to the base model performance, with the model with RAG capabilities performing well on the ABT Q/A and the model achieving a higher score in toxicity type: human than toxicity type: rat. However, we can also observe a decrease in evaluation score for the agent-focused Q/A prompts.

We can observe that the addition of RAG, using our implementation, is consistent with less variance in evaluation score for the toxicity type prompts on average; however, this does not necessarily correspond to an improvement in answer assertion coverage for those categories, as the level 2 runs achieved scores lower than those of the base model across all three rounds.

### Agent with MCP tool integration and comparison to RAG/base model

Figure 5 and Table 2 outline agentic performance across all four prompt categories. On average, MCP runs had slightly higher scores than base model runs on the ABT Q/A prompts. On average, the level 3 agents also achieved higher scores on the agent-focused Q/A when compared to the base GPT-5.5 model. Level 3 scores were similar to those of the baseline scores for the toxicity type: human prompts and were noticeably lower than the baseline scores for the toxicity type: rat prompts.

When compared to the level 2 runs, the level 3 runs performed similarly on average on the ABT Q/A prompts. The level 3 runs achieved slightly higher scores with for both toxicity type prompt categories on average compares to the level 2 runs. Finally, the level 3 runs achieved higher average scores for the agent-focused Q/A prompts when compared to the level 2 runs.

## Discussion

*ToxPipe* is a modular, fully open-source software ecosystem designed to make LLM capabilities accessible and manageable for toxicological research. It combines a ChatGPT-like interface, a standardized model-routing layer, visual workflow tools, and administrative and monitoring services within a unified environment for researchers, developers, and organizations. Through a single interface and common API, users can access models from multiple providers as well as locally hosted open-weight models. *ToxPipe* extends these models with RAG, scientific literature search, and agentic workflows through ToxPipeMCP, which connects them to curated toxicological data, chemical analysis tools, NTP reports, and scientific literature from PubMed. The platform also captures model interactions, tool use, latency, token consumption, and costs to support oversight and reproducible development. Distributed under an MIT license as a collection of linked Docker containers, *ToxPipe* provides a deployable and customizable foundation for using natural-language interactions to integrate, summarize, and interpret toxicological information.

### Prompt observations

The high scores observed over all three levels on the ABT Q/A prompts may be due to the limited answer format (multiple-choice) heavily restricting the domain of possible answers for the model’s response. While this hypothesis was not explicitly accounted for in our experimental design, heightened LLM performance on multiple-choice questions compared to open questions has been observed in other studies (Singh et al., 2025). Additionally, the ABT Q/A dataset contains data from 2000 to 2007, which is well within the generator model’s training data range: it is not unlikely that these data are included in GPT-5.5’s training corpus. It is important to keep in mind that the ABT Q/A scores may be affected by contamination if these items were included in the model’s training corpus, which could produce these inflated scores; however, this too was not explicitly tested for.

### RAG observations

We attempted to improve the data availability problem of limited or out-of-date toxicological data by introducing bespoke RAG and tool resources to popular LLMs. We set up a very basic RAG implementation with primarily default settings, as we weremore interested in exploring the possibilities of this technology rather than using it for regulatory judgment; however, this introduced the following issues into the RAG pipeline:

1. The embedding process created a lot of noise or “garbage” data extracted from the input PDF documents.
2. The models used in the RAG pipeline, GPT-4o and text-embedding-ada-002, are fairly old and have been surpassed by newer text generation and embedding models, respectively.
3. The text splitting chunk size is large enough to the point where it frequently did not activate due to a document’s pages being smaller than the chunk size; therefore these pages wereembedded in their entirety into a single node per page.
4. The embedding pipeline used an older PDF extractor, which may have introduced imperfections in extracting data from the documents.
5. We iteratively developed the embeddings database, but we failed to fully clear the collection of embeddings as we regenerated it throughout development, so some documents are processed and stored multiple times.

These issues may have accounted for the observations at level 2; however, more work is needed to determine if this is the case. Additional future work on *ToxPipe* will focus on retuning our RAG pipeline’s parameters and cleaning the underlying data before rerunning. We aim to also standardize the pipeline so the same model is used for both embedding creation and retrieval. We can glean from this exercise that RAG may not be a “one-size-fits-all” solution: the effectiveness of our RAG implementation may depend on the quality of the underlying embeddings database and the ease with which it can be searched. Additionally, running the RAG embedding process, particularly on a large number of documents, can be extremely slow.

### Agent with tool observations

We observed mixed performance in agents compared to their base model counterparts. When given prompts specifically designed to encourage tool use, the mean model score increased; however, the mean score decreased in the toxicity type: rat prompts, many of which fall outside the domains covered by the tools’ data. One hypothesis for this behavior is that the model may prioritize data returned by MCP tools when those tools are available, even if they are detrimental to answering a prompt; however, this claim was not tested in this study. It may be possible to remedy this with more explicit system instructions to the agent and/or a more strictly defined flow between agent states to enforce/discourage the use of tools and training data as needed. An alternate hypothesis for these observations is that certain tools are capable of returning large amounts of data and overwhelming the context window, which can cause blank or malformed model responses. This was also not tested in this study, and future work should be taken to limit tool output to be more targeted and precise. Overall, differences in level 3 performance from the baseline may possibly be due to generation, tool call, and/or availability inconsistency, but our experimental design does not distinguish between these sources.

Throughout our testing, we informally observed issues when using MCP tools. These problems introduced several new design considerations that should be accounted for in future work such as: reliance on external services that may be unavailable, overly long or poorly formatted tool output, slow computations, model responses being flagged for content violations, and difficulty deciding which tools to call including the omission of potentially helpful tools. It is important to acknowledge that using LLMs with tools can be an inconsistent process, so care should be taken to run models multiple times and note any inconsistencies in the outputs.

### Evaluation process limitations and caveats

First, it is important to note that LLM responses, tool calls, and evaluations of responses can vary between runs, so users of ToxPipe should expect inconsistencies and variability in model output. In the future, it may be beneficial to perform repeat evaluations on the same response with each of the evaluator models.

The LLM-as-a-judge evaluation process relies entirely on the judgment of an LLM to identify semantic connections in model responses, a task for which these LLMs have not been independently validated in this study. Under our current experimental design, the LLM-as-a-judge method is really only capable of informing us if the generator model’s response to a given prompt aligns to the corresponding predefined assertions based on some heuristic internal to each evaluator model and unknown to the researcher.

Additionally, the judging process may subject a given evaluation to the evaluator model’s internal biases. While we recognize that GPT-5.5 may be (intentionally or unintentionally) biased in evaluating responses produced by another instance of GPT-5.5, GPT-5.5 scored similarly to Claude 4.5 Haiku and Gemini 3.5 Flash across all 4 prompt categories. Due to the fact that the evaluation scores only measure assertion agreement, however, these results cannot be used to determine bias across the evaluator models. Conversely, while the results in Table ?? Do not show a large difference in marginal mean score between the evaluator models, this analysis cannot completely rule out all model bias either.

Therefore, the LLM-as-a-judge method may not be the most appropriate strategy for evaluating an LLM-generated response’s truthfulness, as it can only report a response’s assertion coverage, not the accuracy of the statement. Additionally, a lower evaluation score may reflect stricter evaluator criteria rather than lower response assertion coverage, but this was not tested for in this study. Our LLM-as-a-judge scoring implementation was also not designed to penalize responses that include additional information beyond the accepted assertion(s). As long as the model’s response is deemed to include all of the required assertions, the run will be given a perfect evaluation score, even if it includes additional irrelevant, contradictory, or incorrect information. For a multiple-choice style prompt like the ABT Q/A prompts, the model could hypothetically get a perfect score by selecting every multiple choice answer every time. This illustrates a too-forgiving pass criterion that may not be appropriate in settings where precision is paramount.

The evaluated agentic Q/A and toxicity type: rat categories are very small, with only 10 and 12 different prompts, respectively. Therefore, even a single prompt can disproportionately affect the categorical results.

### Architectural additions to institutional LLMs

*ToxPipe* is configured to offer a centralized, containerized environment of LLMs, interfaces, data resources, and monitoring tools. All models are accessed through the LiteLLM interface using the Azure OpenAI API specification, making model access homogeneous and centralized. LiteLLM and Langfuse offer simple cost/usage tracking, tracing of prompts and tool calls, and better observability. While open-weight models do not always expose their source code or training corpora, they may be fine-tuned and deployed/managed locally for increased privacy and transparency. Thus, *ToxPipe* offers multiple features:

- ready access to a range of approved models
- increased modularity and customization for model setup
- open-source code base and transparency with support for open-weight, locally hosted models
- quick access to popular toxicological data resources that may supplement the information available to institutional AI models

### Dataset integration

In our implementation, *ToxPipe* used the available RAG and MCP technology to add context to model responses. However, the utility of our provided resources may be limited by the quantity and quality of the underlying data and how it is stored.

### Future work

The following are some possible avenues for generally improving model performance in future updates:

- Additional and more selective data source curation
- Different or more careful language use in prompting, particularly in the system instructions
- Use of newer, more robust models with larger context windows, up-to-date training data, and better decision-making capabilities
- Cleaning the data returned from the tools before passing it to the agent for interpretation
- Delegating different steps of a workflow to sub-agents to avoid unnecessarily consuming the context window
- Expanding functionality for analyzing novel and poorly characterized chemical data
- Retuning the RAG embeddings database

LLM technology is rapidly improving and expanding, so developing *ToxPipe* has been an important exercise for our group to evaluate how to best use this technology in the context of toxicological research. We observed inconsistent results in our evaluations using *ToxPipe*’s current RAG and MCP integration, particularly in the lower-assertion-coverage rat toxicity stratum. More refinement and testing is necessary to determine whether our LLM pipeline is suitable for real-world applications such as chemical risk assessment, regulatory decision-making, and public health.

### Study limitations

This research is limited in that domain-specific improvements to existing AI models are only additive; that is, we have only supplemented existing instances of GPT-5.5 with external information without affecting weights or the model’s original training corpus. *ToxPipe* does not include fine-tuning of models on toxicology-specific data at this stage. Additionally, the scope is restricted to predefined toxicological datasets and publicly available toxicological reports. While *ToxPipe* provides a framework for integrating other types of datasets, such integrations are outside the scope of this initial evaluation. Further development will be needed to extend *ToxPipe*’s functionality to broader toxicological challenges, such as open-ended question answering or the analysis of novel, sparsely-documented, and/or highly complex chemicals not included in the present dataset.

Future evaluation work should include expert adjudication. We should also refine our evaluation metrics to account for assertion-level agreement when comparing evaluator model output. Furthermore, to improve reliability and consistency of the evaluations in the future, we could use a different (non-language) model to identify semantic relationships between concepts in the model responses. We could also use multiple models from different sources to evaluate the responses and synthesize their outputs into a consensus statement.

The logical connections in an LLM’s “thought process” should not be interpreted as evidence of true “understanding” of toxicological data connections. While GPT-5.5 scored higher on the high-assertion human toxicity stratum versus the lower-assertion rat toxicity stratum, the imbalance of the number of evaluated prompts between the two strata make it difficult to specifically attribute this scoring difference to species-based data density.

*ToxPipe*’s security was also not formally evaluated in this study. Users should not upload proprietary or sensitive data to *ToxPipe*’s services or models. A properly access-controlled, fully local deployment of *ToxPipe* may be appropriately secure for working with such data, but this capability is outside of the scope of this study.

Current concerns about reproducibility, reliability, and public trust in LLM technology may limit the use of *ToxPipe* in regulatory decision-making. Despite these limitations, we hope that the documentation and transparency of the *ToxPipe* stack can bring further attention to what AI tools and techniques are currently available for use in predictive toxicology applications and can facilitate further improvements to toxicological report generation.

The results collected in this study are not appropriate for judging generator model response accuracy or agreement with expert opinion, only a response’s alignment with predefined assertions. Furthermore, the results cannot support any statement on inter-model agreement among the evaluator models, as the evaluator models can achieve similar average scores while disagreeing at the assertion level.

## Conclusion

*ToxPipe* applies recent advancements in artificial intelligence for synthesizing unstructured toxicological information into coherent narratives. While the platform has the potential to improve the efficiency and effectiveness of toxicological research, *ToxPipe*’s current offerings are inconsistent. The addition of our RAG and MCP deployments did not provide a consistent, observed increase to GPT-5.5’s response assertion coverage relative to its baseline performance. Additionally, MCP tools can introduce external dependencies into an AI pipeline that make management and reliable access challenging. Because the scoring method was not validated by expert opinion, the benchmark datasets were uneven, and our RAG pipeline was under-tuned, these results should not be interpreted as statements on model response accuracy and truthfulness, the effectiveness of MCP or RAG as a whole, or the fitness of LLMs for regulatory use.

As newer models and adjacent technology are released to the public, we will integrate them into the *ToxPipe* ecosystem to fully take advantage of any benefits they may provide. However, future development should ensure that the data accessible to *ToxPipe* are up-to-date and that the tools and embeddings available to *ToxPipe* are properly tuned and configured to avoid issues like the ones we identified in our implementation during the evaluations.

## Data Availability

The datasets presented in this study can be found in online repositories. The names of the repository/repositories and accession number(s) can be found in the article/supplementary material.
